# T follicular helper cells and T follicular regulatory cells in autoimmune diseases

**DOI:** 10.3389/fimmu.2023.1178792

**Published:** 2023-04-28

**Authors:** Jingjing Qi, Chang Liu, Ziran Bai, Xia Li, Genhong Yao

**Affiliations:** ^1^ Department of Immunology, College of Basic Medical Science, Dalian Medical University, Dalian, Liaoning, China; ^2^ Department of Rheumatology and Immunology, Dalian Municipal Central Hospital, Dalian, Liaoning, China; ^3^ Department of Rheumatology and Immunology, The Affiliated Drum Tower Hospital of Nanjing University Medical School, Nanjing, Jiangsu, China

**Keywords:** T follicular helper cells (TFH cells), T follicular regulatory cells (TFR), autoimmune diseases, rheumatoid arthritis, systemic lupus erythematosus, Sjögren’s syndrome, type 1 diabetes, multiple sclerosis

## Abstract

T follicular helper (Tfh) cells are heterogeneous and mainly characterized by expressing surface markers CXCR5, ICOS, and PD-1; cytokine IL-21; and transcription factor Bcl6. They are crucial for B-cell differentiation into long-lived plasma cells and high-affinity antibody production. T follicular regulatory (Tfr) cells were described to express markers of conventional T regulatory (Treg) cells and Tfh cells and were able to suppress Tfh-cell and B-cell responses. Evidence has revealed that the dysregulation of Tfh and Tfr cells is positively associated with the pathogenic processes of autoimmune diseases. Herein, we briefly introduce the phenotype, differentiation, and function of Tfh and Tfr cells, and review their potential roles in autoimmune diseases. In addition, we discuss perspectives to develop novel therapies targeting Tfh/Tfr balance.

## Introduction

Autoimmune diseases refer to a category of diseases with high prevalence (7%–9%) in the general population, causing considerable mortality. Autoimmune diseases can be initiated by immune responses mistakenly targeting an individual’s cellular components, resulting in tissue damage and organ dysfunction. According to the tissues involved, they can be categorized as organ-specific diseases, including type 1 diabetes (T1D) and multiple sclerosis (MS), and multiple organs involving systemic diseases, including rheumatoid arthritis (RA), systemic lupus erythematosus (SLE), Sjögren’s syndrome (SS), and granulomatosis with polyangiitis (GPA) (1, 2). Many autoimmune diseases are characterized by autoantibody production. Autoantibodies promote disease pathogenesis by forming immune complexes, which mediate tissue inflammation and damage by activating complement and effector cells (3). Helper T cells play crucial roles in the pathogenesis of autoimmune diseases by secreting immune mediators and helping B cell-mediated long-lived humoral immunity development (4).

T follicular helper (Tfh) cells are a CD4^+^ T-cell subset that promotes germinal center (GC) formation, antibody affinity maturation, and memory B-cell generation (5). Recent studies have found a specialized subset of T regulatory (Treg) cells named T follicular regulatory (Tfr) cells, which can negatively regulate GC responses (6). Here, we review the established phenotype and function of Tfh and Tfr cells and their roles in the pathogenesis of autoimmune diseases, and highlight the potential therapies targeting these cells.

## Phenotypes of Tfh and Tfr cells

**Figure 1:**
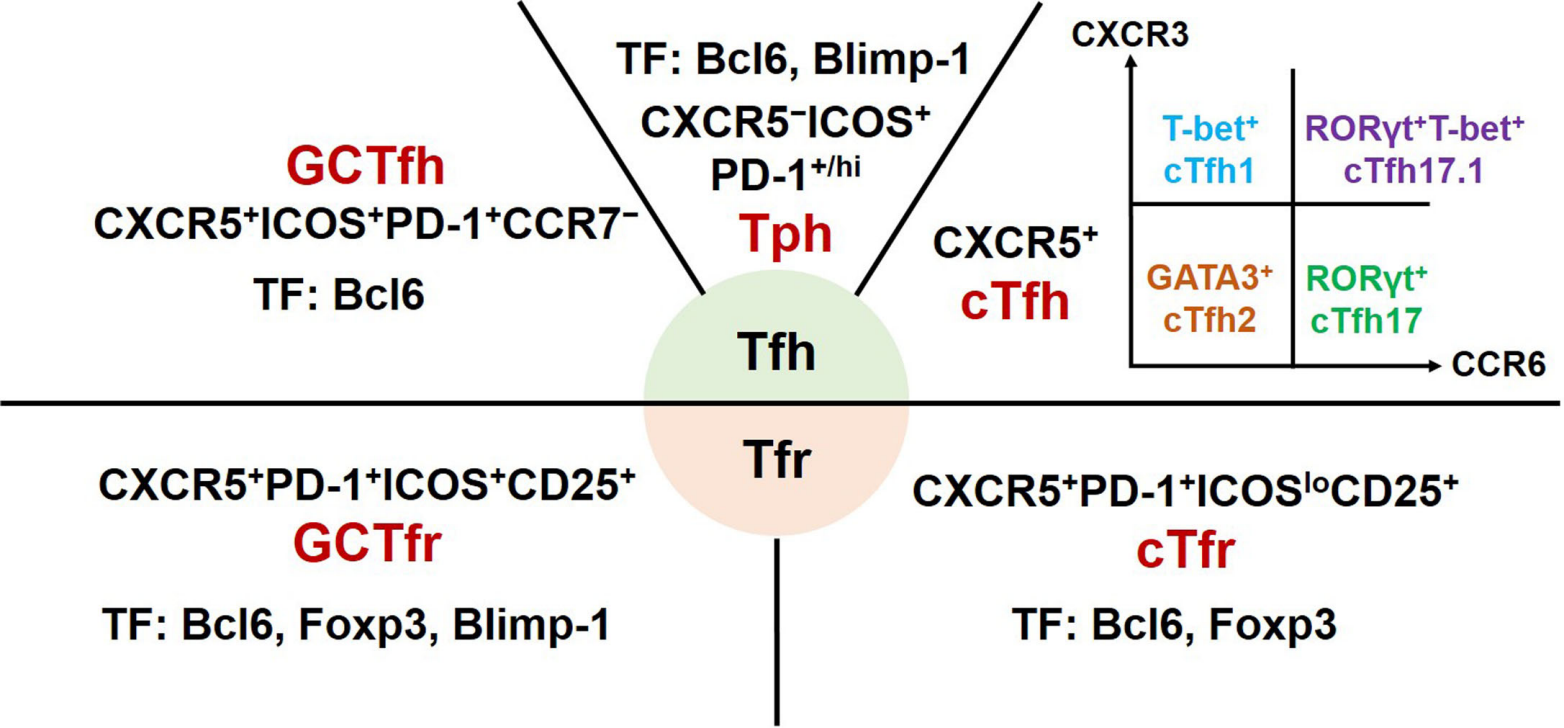
Phenotype and transcription factors of Tfh effector cell subsets and Tfr cells. GC, germinal centers; Tfh, T follicular helper; cTfh, circulating Tfh; Tph, T peripheral helper; Tfr, T follicular regulatory; cTfr, circulating Tfr; CXCR, chemokine receptor; ICOS, inducible T-cell co-stimulator; PD-1, programmed cell death protein-1; Bcl6, B cell lymphoma 6; Foxp3, forkhead box P3; GATA3, GATA-binding protein 3; ROR, retinoid-related orphan receptor; Blimp-1, B lymphocyte-induced maturation protein 1; TF, transcription factors.

Tfh cells are a heterogeneous subset of CD4^+^ T cells (**Figure 1**). Initial studies of experimental animal models revealed that Tfh cells mainly reside in GCs of secondary lymphoid organs. These canonical GC Tfh cells are characterized by expressing transcription factor B-cell lymphoma 6 (Bcl6), CXC-chemokine receptor 5 (CXCR5), inducible T-cell co-stimulator (ICOS), and programmed cell death protein-1 (PD-1) (7, 8). The clinical studies mainly focus on Tfh cells from the peripheral blood of patients. Circulating Tfh (cTfh) cells share phenotypic surface markers CXCR5, ICOS, and PD-1 with GC Tfh cells. Based on the expression of CXCR3 and CCR6, cTfh cells are further divided into four major subsets: CXCR3^+^CCR6^−^ cTfh1, CXCR3^−^CCR6^−^ cTfh2, CXCR3^−^CCR6^+^ cTfh17, and CXCR3^+^CCR6^+^ cTfh17.1 cells. These cTfh cell subsets share common precursors with their equivalent helper T cell (Th) 1, Th2, Th17 or Th1, and Th17 cells, respectively (9–11).

Tfr cells, a subset of regulatory T cells, were described as sharing phenotypic markers with conventional Treg cells (CD4^+^CD25^+^Foxp3^+^) and Tfh cells (12, 13). Subsequent studies reported that circulating Tfr (cTfr) cells should be defined as CD4^+^CXCR5^+^Foxp3^+^ T cells and tissue-resident Tfr cells fully expressing CXCR5, ICOS, and PD-1 (14, 15). In addition, Tfr cells as a heterogeneous subset may express different phenotypic characteristics at different development stages in distinct inflammatory milieus (10).

## Differentiation of Tfh and Tfr cells

### Transcription factors

Bcl6 is the essential transcription factor for Tfh cell differentiation by controlling the expression of CXCR5, interleukin-6 receptor (IL-6R), IL-21, and IL-21R in naïve CD4^+^ T cells. Bcl-6 can also inhibit the effects of the transcription factors T-bet for Th1 cells, GATA3 for Th2 cells, and RORγt for Th17 cells by downregulating the expression of B lymphocyte-induced maturation protein 1 (Blimp-1) (4, 11, 16, 17). In particular, CXCR5^+^ cTfh cells, with effector memory phenotype, do not express Bcl6 owing to the lack of persistent antigen stimulation (18). Upon antigen re-encounter, these pre-Tfh cells can rapidly differentiate into mature GC Tfh cells (19, 20).

Unlike Tfh cells, Tfr cells can differentiate from thymic-derived natural Treg cells and peripheral Treg cells (12, 13, 21). These precursor cells differentiate into Tfr cells requiring transcription factor Bcl6. Similar to Tfh cell differentiation, ICOS signaling promotes the development of Tfr cells by upregulating Bcl6 expression (22). Compared with GC Tfr cells, cTfr cells expressed a similar level of CXCR5, but a lower level of ICOS (15). Blimp-1 is necessary for the Treg-like suppressive function and homing into GCs of Tfr cells (13, 23).

### Cytokines

**Figure 2:**
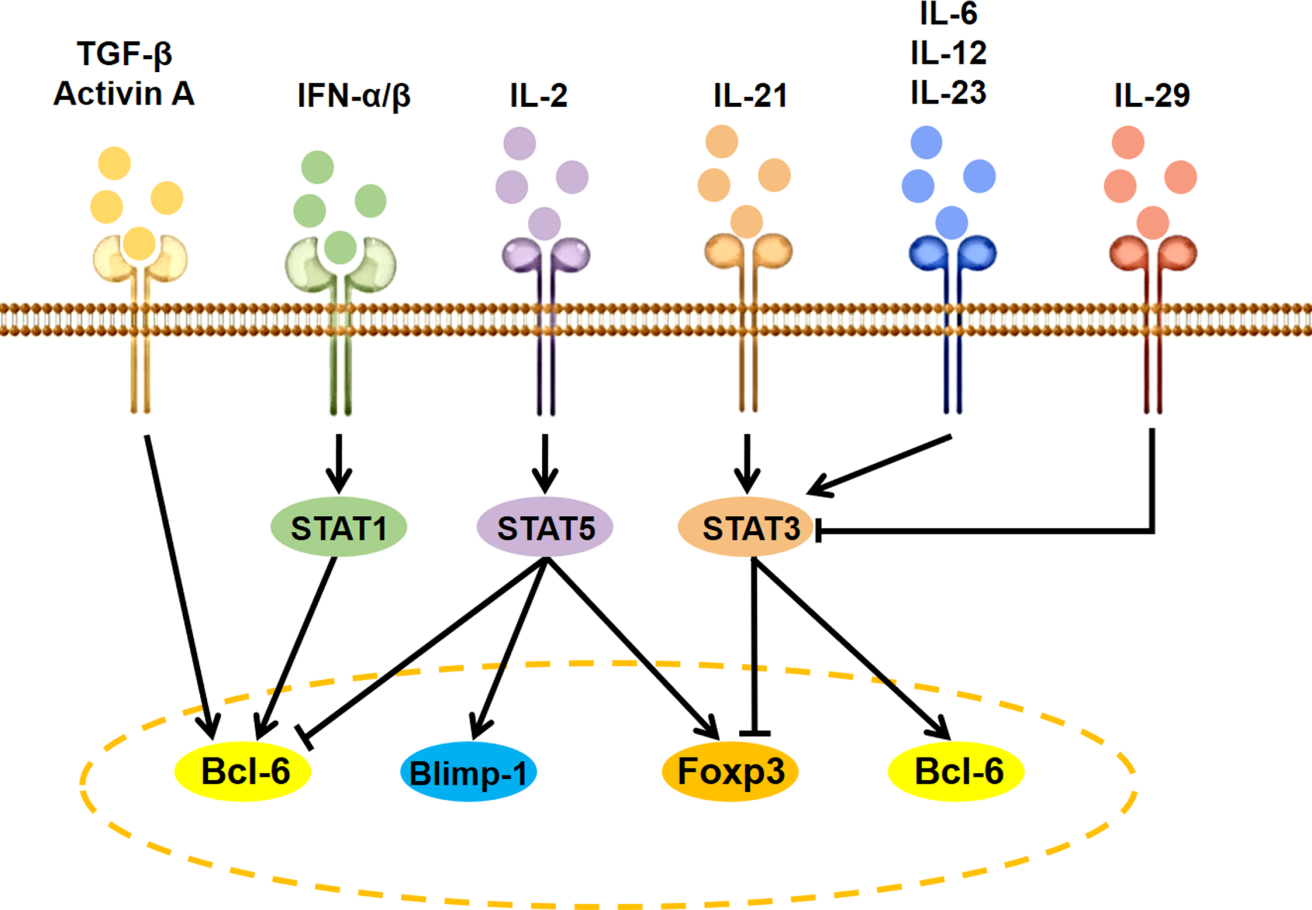
Signaling modulation of Tfh and Tfr cell differentiation. IL, interleukin; TGF, transforming growth factor; IFN, interferon; STAT, signal transducers and activators of transcription; Bcl6, B cell lymphoma 6; Foxp3, forkhead box P3; Blimp-1, B lymphocyte induced maturation protein 1.

Besides antigen stimulation to activate T-cell receptor (TCR) signaling and costimulatory signals via CD28 and ICOS, the expression and activity of Bcl6 are regulated by several specific cytokine-initiated cell-intrinsic signaling cascades (**Figure 2**). The IL-2–STAT5 pathway inhibits GC Tfh cell formation by inducing Blimp-1 to suppress Bcl6 expression (24). The IFN-α/β-STAT1 pathway contributes to CXCR5 and PD-1 expression in naive mice CD4^+^ T cells by inducing Bcl6 (25). Co-stimulation of IFN-α and IL-2 can convert CXCR5^+^PD-1^+^ cTfh cells to CXCR5^−^PD-1^+^ T peripheral helper (Tph) cells through promoting the binding of STAT5 to the Bcl6 locus at the expense of STAT1 (26). IL-12 is the most efficient cytokine inducing human naive CD4^+^ T cells to express IL-21. The IL-21–STAT3 pathway can promote GC Tfh cell differentiation by inducing Bcl6 expression and antagonizing IL-2 signals (27, 28). IL-23, as the substitute for IL-12, can also induce IL-21 expression and human cTfh cell differentiation *in vitro* (29). The IL-6 is a potent inducer for IL-21 expression and GC Tfh cell differentiation of naive murine CD4^+^ T cells by inducing STAT3 phosphorylation (27, 30). Of special interest is that IL-29 may suppress cTfh differentiation through decreasing STAT3 activation-induced Bcl6 expression (31). In addition, TGF-β and TGF-β superfamily member Activin A seems to be important for human, but not murine, Tfh cell differentiation (29, 32).

IL-2, as a positive factor for Treg cell differentiation and a negative factor for Tfh cell differentiation (33), can facilitate cTfr cell development by upregulating Foxp3 and Bcl6 expression (34, 35). IL-21 and IL-6 as the positive factor for Tfh cell differentiation can inhibit Tfr cell development by suppressing Foxp3, TGF-β, or CD25 expression via activating the STAT3 signaling pathway (36–38), while STAT3 deficiency decreased both Tfr and Tfh cell differentiation (39).

## Functions of Tfh and Tfr cells

Tfh cells are a subset of effector T cells that can assist B-cell maturation, high-affinity antibody production, and memory B-cell development in GCs (**Table 1**). CXCR5, a receptor for chemokine ligand CXCL13, can guide Tfh cells into GCs and interact with B cells. It has been shown that a high level of CXCR5 combined with a low level of CCR7 is required for T cells to migrate to the T–B border (10). Within GCs, Tfh cells interact with B cells relying on ICOS-ICOSL, CD40L-CD40, and TCR-peptide-MHC II, which induces IL-4, IL-21, and Bcl-6 expression in Tfh cells and promotes B-cell activation (4, 40, 41). Furthermore, IL-21 binding to IL-21R on Tfh and B cells can facilitate their proliferation and differentiation to maintain GC responses (42, 43), while PD-1 signals can restrain GC Tfh cell proliferation by inhibiting ICOS signaling, avoiding excessive B-cell proliferation and antibody production (53).

**Table 1:** Functional factors and roles in B-cell activation of Tfh and Tfr cells.

Tfh cell subsets	Functional factors	Functions	References
GC Tfh	IL-21, IL-4, CD40L	Help for B-cell maturation, high-affinity antibody production, antibody class switching, and memory B-cell development.	(4, 8, 10, 40–43)
cTfh			
cTfh1	IL-21, IFN-γ, IL-10	Help for memory B-cell differentiation into plasma cells.	(8, 10, 19, 20, 44–47)
cTfh2	IL-21, IL-4, IL-5, IL-13	Help for IgM, IgG, IgA, and IgE secretion.	
cTfh17	IL-21, IL-17, IL-22	Help for IgM, IgG, and IgA secretion.	
cTfh17.1	IL-21, IL-17, IL-22, IFN-γ	Help for IgM, IgG, and IgA secretion.	
Tph	IL-21, SLAMF5, CXCL13	Help for B-cell function with a migratory program targeting inflamed tissues and recruitment of Tfh and B cells to inflamed tissues.	(26, 48)
GC Tfr	CTLA-4, IL-10, TGF-β, granzyme B	Strong suppression on Tfh-cell and B-cell responses in GC.	(13, 15, 23)
cTfr	CTLA-4, IL-10, TGF-β, granzyme B	Less suppression of Tfh-cell and B-cell responses in GC.	(6, 49–52)

GC, germinal centers; Tfh, T follicular helper; cTfh, circulating Tfh; Tph, T peripheral helper; Tfr, T follicular regulatory; IFN, interferon; IL, interleukin; TGF, transforming growth factor; SLAMF, signaling lymphocyte activation molecule family; Ig, immunoglobulin; CTLA-4, cytotoxic T-lymphocyte antigen 4.

Upon re-encountering antigen, extrafollicular cTfh cells rapidly differentiate into mature GC Tfh cells and are guided by CXCR5-CXCL13 to GCs in secondary lymphoid organs (19, 20). cTfh1 cells express high levels of IFN-γ; cTfh2 cells express high levels of IL-4, IL-5, and IL-13; and cTfh17 cells express high levels of IL-17 and IL-22. All these cytokines help class-switching of GC B cells (10, 44). Studies reported that distinct cTfh cell subsets, except cTfh1 cells, are prone to induce naive B cells to differentiate into plasma cells secreting different classes of immunoglobulins (8, 45).

CXCR5 can also guide Tfr cells to migrate to GCs (6). GC Tfr cells show durable and persistent suppression of high-affinity autoantibody production by inhibiting glucose metabolism in B cells (49). GC Tfr cells may limit GC reactions by physically interrupting Tfh–B cell recognition via cytotoxic T-lymphocyte antigen 4 (CTLA-4) competitive binding with the co-stimulatory molecules on GC B cells (49, 50). Moreover, similar to Treg cells, Tfr cells control Tfh- and B cell-mediated immune responses by secreting IL-10, TGF-β, and granzyme B (51). However, compared to GC Tfr cells, the cTfr cells showed less suppressive function on B-cell responses (52).

## Involvement of Tfh and Tfr cells in autoimmune diseases

The interactions between B and Tfh cells are crucial for autoantibody production, which is the hallmark of autoimmune diseases. Considerable evidence has revealed that the imbalance of Tfh and Tfr cells is involved in the development of autoimmune pathology (**Table 2**).

**Table 2:** Human autoimmune diseases associated with aberrant Tfh-cell function. .

Autoimmune diseases	Tfh-cell subsets	Correlated pathology
Rheumatoid arthritis	Increased CXCR5^+^PD-1^+^ Tfh cells (54–56); cTfh2 and cTfh17 cells (57); CXCR5^−^PD-1^hi^ Tph cells (48, 58); Decreased CXCR5^+^CD25^+^CD127^lo^ cTfr cells (59–61); Decreased Tfr/Tfh ratio (59–61).	Tph cells contribute to chronic autoimmune phenomena at the inflammatory foci (48, 58); Tfr/Tfh ratio is negatively correlated with CRP, ESR, ACPA, and DAS28 index (59–61).
Systemic lupus erythematosus	Increased NLRP3 active Tfh cells (62); CXCR5^−^PD-1^hi^ Tph cells (26, 63, 64); CXCX5^−^PD-1^hi^CXCR3^+^ Tfh-like cells in blood and the tubulointerstitial areas (65); CCR6^+^IL7R^+^IL-10^+^ Tfh-like cells in peripheral blood and LN (66); CXCR5^+^FOXP3^+^ Tfr cells (67); expression of PD-1 on CXCR5^+^FOXP3^+^ Tfr cells (68); Decreased CXCR5^+^CD25^+^CD127^lo^ Tfr cells (69); expression of Foxp3, CTLA4, and IL-2 receptor on CXCR5^+^FOXP3^+^ Tfr cells; Increased/Decreased Tfr/Tfh ratio (69).	Tfh cells with active NLRP3 inflammasome are essential for optimal humoral responses and GC formation (62); Tph cells contribute to B-cell responses via IL-21 (26, 63, 64); CXCX5^−^PD-1^hi^CXCR3^+^ Tfh-like cells provide B-cell help, independently of IL-21, by producing IL-10 and succinate (65). IL-10-producing CCR6^+^IL7R^+^ Tfh-like cells are associated with anti-dsDNA antibodies and promoted B-cell IgG production (66). Tfr cell frequency and Tfr/Tfh ratio are negatively correlated with serum IL-21 concentration, anti-dsDNA antibody levels and disease activity (69).
Sjögren’s syndrome	Increased CXCR5^+^ICOS^+^PD-1^+^ Tfh cells (70, 71); CCR7^lo^PD-1^hi^ cTfh cells (72); Tfh1, Tfh2, and Tfh17 cells in SG (71, 73); CCR9^+^ Tfh-like cells in peripheral blood and LSG (74); CXCR5^+^PD1^+^ICOS^+^Foxp3^−^ Tfh cells and CXCR5^−^PD1^hi^ICOS^+^Foxp3^−^ Tph cells in peripheral blood and SG (75, 76); FoxP3^+^CXCR5^+^ Tfr cells (71, 77); Decreased CXCR5^+^FoxP3^+^CD25^+^ Tfr cells (78); Decreased Tfr/Tfh ratio (78).	IL-21^+^ or ICOS^+^ Tfh cells are positively correlated with transitional B cells, plasmablasts, and plasma cells (70, 79); cTfh cells are positively correlated with disease activity scores and plasma cell percentages (72). CCR9^+^ Tfh-like cells promote IgG production and display higher levels of IFN-γ, IL-17, IL-4, and IL-21 than CXCR5^+^ Tfh cells with antigen or IL-7 stimulation (74).
Granulomatosis with polyangiitis	Increased CXCR5^+^PD-1^+^ cTfh cells (80), CD4^+^IL-21^+^, CD4^+^IL-21^+^IL-17A^+^, and CD4^+^BCL6^+^ T cells (81).	CD4^+^IL-21^+^ and CD4^+^BCL6^+^ T cells are elevated only in ANCA-positive GPA patients (81).
Multiple sclerosis	Increased cTfh1 and CXCR3^+^CCR6^+^ cTfh17.1 cells in CNS (46); CD4^+^IL-21^+^ T cells in the lesions (82), CCR7^+^ICOS^+^ cTfh cells, cTfh17 cells and cTfh17.1 cells (83–87); CXCR5^+^PD-1^+^ Tfh cells in CSF (88); Decreased cTfh1 cells and cTfh2 cells (83–87); CXCR5^+^CD25^+^PD-1^+^ FoxP3^+^/CD127^−^ or CXCR5^+^CD25^hi/+^ CD127^dim/−^ Tfr cells in blood and CSF (85–87, 89); Decreased Tfr/Tfh ratio (87, 90).	cTfh cells secrete high level of IL-21 (85); CCR7^+^ICOS^+^ cTfh cells are positively correlated with disease activity scores, the levels of IL-21and IgG in plasma and CSF (86); cTfr cells secrete low level of IL-10 (85); Tfr cells exhibit reduced suppressive capacity in blood and CSF (85–87, 89); Tfr/Tfh ratio are negatively correlated with the levels of IgG in serum and CSF (87, 90).
Type 1 diabetes	Increased Tfh cells; Tph cells (91–95); Tfh1 cells in pancreas (96); Decreased Tfr cells in the peripheral blood, spleen and pancreatic lymph nodes (97, 98); Decreased Tfr/Tfh ratio (97, 98).	Tfh and Tph cells are associated with T1D progression by producing IL-21 and recruiting and activating B cells (91–95). Tfh1 cells promote T1D development (96). Tfr cells show attenuate suppressive ability (97, 98).

GC, germinal centers; Tfh, T follicular helper; cTfh, circulating Tfh; Tph, T peripheral helper; Tfr, T follicular regulatory; ICOS, inducible T cell co-stimulator; PD-1, programmed cell death protein-1; Foxp3, forkhead box P3; Bcl6, B-cell lymphoma 6; CRP, C-reactive protein; ESR, erythrocyte sedimentation rate; ACPA, serum anti-cyclic citrullinated peptide antibodies; DAS28, disease activity score-28; Ig, immunoglobulin; LN, lymph nodes; SG, salivary glands; LSG, labial salivary glands; ANCA, antineutrophilic cytoplasmic autoantibody; CNS, central nervous system; CSF, cerebrospinal fluid; T1D, type 1 diabetes.

## Rheumatoid arthritis

RA is a common systemic autoimmune disease mainly characterized by chronic inflammation affecting the joints and other organs (99). Studies indicated that CXCR5^+^ Tfh cells were present in the B-cell area of lymphoid tissue from early RA patients, and both CXCR5^+^ and CXCR5^+^PD-1^+^ cTfh cell proportions were higher in untreated early RA patients than in healthy controls (HC) (54–56). The increased circulating plasmablasts in RA patients promoted CXCR5^+^ICOS^+^ cTfh cell differentiation via IL-6 production (100). Furthermore, in patients with low or high active RA, the frequencies of cTfh1 cells were comparable with those in HC. However, the frequencies of cTfh2 and cTfh17 cells were higher than those in HC. Patients with high active RA had more cTfh2 and cTfh17 cells than patients with low active RA (57). OX40 expressed cTfh, especially cTfh17 cells, were increased, and negatively correlated with autoantibody sialylation in RA patients (101).

A novel CXCR5^−^PD-1^hi^ Tfh cell population in the synovial tissues and peripheral blood of seropositive RA patients was defined as Tph cells. Tph cells were increased only in patients with high active RA and might contribute to chronic autoimmune phenomena at the inflammatory foci (48, 58). Adiponectin (AD) promoted fibroblast-like synoviocytes producing IL-6 to enhance CXCR5^+^PD-1^+^ cTfh cell responses in RA patients. Intra-articular injection of AD aggravated synovial inflammation with increased Tfh cells in the joint tissue of collagen-induced arthritis (CIA) mice (100).

Moreover, compared with HC, RA patients showed decreased CXCR5^+^CD25^+^ CD127^lo^ or CXCR5^+^Foxp3^+^ cTfr cells and Tfr/Tfh ratio. The ratio of Tfr/Tfh was negatively correlated with C-reactive protein (CRP), erythrocyte sedimentation rate (ESR), serum anti-cyclic citrullinated peptide antibodies (ACPA), and disease activity score-28 (DAS28) index of RA patients (59–61). Studies on targeting Tfh and Tfr cells revealed that CTLA-4-Ig, iguratimod, abatacept, low-dose IL-2, and alcohol consumption could ameliorate RA by inhibiting Tfh cell responses and restoring the Tfr/Tfh balance (102–107).

## Systemic lupus erythematosus

SLE is a prototypic autoimmune disease with aberrant activation of T and B cells. Multiple serum autoantibodies against nuclear antigens lead to systemic tissue damage (108). According to studies, the increased cTfh cells and serum IL-21 were associated with the pathogenesis of SLE patients (109). Furthermore, in SLE patients, the percentages of cTfh1 and cTfh2 cells were comparable with those in HC, and the percentages of cTfh17 cells were higher than those in HC (110, 111). A population of Tfh cells with active NLRP3 inflammasome was increased and essential for optimal humoral responses and GC formation in SLE patients and mice (62). Circulating CXCR5^−^PD-1^+/hi^ Tph cells were increased significantly, which stimulated B-cell responses via secreting IL-21 in SLE patients (26, 63, 64). CXCX5^−^PD-1^hi^ CXCR3^+^ Tfh-like cells expanded in blood and the tubulointerstitial areas of SLE patients, providing B-cell help, independently of IL-21, by producing IL-10 and succinate (65). IL-10-producing CCR6^+^IL7R^+^ Tfh-like cells lacking Bcl6 expression were elevated in peripheral blood and lymph nodes of SLE patients, and these cells were associated with the presence of pathogenic anti-dsDNA antibodies in SLE patients and promoted B-cell IgG production *ex vivo* (66).

Chronic type I IFN production plays a pathogenic role in SLE patients (112). Studies found that type I IFN signals inhibited Tfh cell expansion, but induced Tph cell generation and IL-21 and IFNγ production in Tfh cells by activating STAT4 in lupus mice (113, 114). Circulating immunogenic self-DNA in SLE patients could induce IL-17^+^ Tfh cell expansion via RORγt supporting IgG anti-dsDNA responses (115). OX40L (a TNF superfamily ligand) on myeloid antigen-presenting cells induced human naive and memory CD4^+^ T cells to express Tfh-associated molecules including CXCR5, CD40L, and IL-21 (116).

Xu et al. found that CXCR5^+^CD25^+^CD127^lo^ cTfr cells and the ratio of Tfr/Tfh were decreased significantly in SLE patients. Both cTfr cell frequencies and Tfr/Tfh ratio were negatively correlated with serum IL-21, anti-dsDNA antibody levels, and disease activity of SLE patients (69). However, another study reported that CXCR5^+^Foxp3^+^ cTfr cells and the ratio of Tfr/Tfh were increased in SLE patients. Although the suppressive capacity of cTfr cells was not altered, the cTfr cell frequencies were positively correlated with auto-antibodies and disease activity scores of SLE patients (67). Kurata et al. found that the frequencies of CXCR5^+^Foxp3^+^ cTfr cells were similar in HC and SLE patients, while the expression of PD-1 on cTfr cells was increased and positively correlated with anti-DNA antibody levels and disease activity scores of SLE patients. These cTfr cells had impaired suppressive function with decreased expression of Foxp3, CTLA4, and IL-2 receptors (68).

Clinical studies showed that methylprednisolone pulse treatment decreased the percentages and absolute number of cTfh cells in SLE patients (117). Dexamethasone treatment reduced the frequencies of cTfh2 cells but increased the percentages of cTfh17 cells in SLE patients (111). *Ex vivo*, IL-2 stimulation downregulated the expression of PD-1 along with the increased expression of Foxp3 and CTLA-4 on cTfr cells (68), and converted memory Tfh cells to cTfr cells by promoting STAT3 and STAT5 phosphorylation in SLE patients (34). *In vivo*, sustained low-dose IL-2 therapy reduced cTfh cells significantly but had little effect on cTfr cells, which resulted in recovered Tfr/Tfh ratio in lupus mice and patients (118, 119). IL-2 therapy might inhibit GC Tfh early development from primed CD4^+^ T cells by inhibiting Bcl6 expression (120).

Mesenchymal stem cells (MSCs) ameliorated lupus symptoms in B6.lpr mice by producing iNOS to decrease CXCR5^+^PD-1^hi^ Tfh cell expansion (121). Baicalin and TLR7 agonist imiquimod treatment could relieve lupus mice by inhibiting Tfh cell differentiation and IL-21 production, and promoting Tfr cell differentiation (122, 123). Cotreatment of soluble OX40L and Jagged-1 (a Notch family ligand) alleviated lupus nephritis via increasing Tfr/Tfh ratio, leading to decreased GC B cells and anti-dsDNA antibody levels in NZBWF1/j mice (124). Research revealed that ATP-gated ionotropic P2X7 receptor stimulation limited the expansion of pathogenic Tfh cells by promoting caspase-mediated pyroptosis in a lupus mouse model. Restoring P2X7 activity may limit the progressive amplification of pathogenic autoantibodies in SLE patients (125).

## Sjögren’s syndrome

SS is a heterogeneous systemic autoimmune disease mainly characterized by exocrine gland dysfunction. Anti-Ro/SSA and anti-La/SSB antibodies are important diagnostic indicators of SS (126). CXCR5^+^ICOS^+^PD-1^+^ cTfh cells were significantly increased in SS patients, especially in anti-Ro/SSA antibody-positive patients (70, 71). Compared with HC, SS patients with a high degree of focal lymphocytic sialadenitis had more CCR7^lo^PD-1^hi^ cTfh cells, which were positively correlated with disease activity scores and plasma cell percentages of SS patients (72). IL-21^+^ or ICOS^+^ cTfh cells were positively correlated with transitional B cells, plasmablasts, and plasma cells in SS patients (70, 79). Although cTfh1, cTfh2, and cTfh17 cells were comparable in the peripheral blood of HC and SS patients, they were increased in minor salivary glands (SGs) of SS patients (71, 73). CXCR5^+^PD1^+^ICOS^+^Foxp3^−^ Tfh cells and CXCR5^−^PD1^hi^ICOS^+^Foxp3^−^ Tph cells were enriched in both SS peripheral blood and salivary gland with GCs (75, 76). Foxp3^+^CXCR5^+^ cTfr cells were increased in SS patients, especially in autoantibody-positive SS patients (71, 77), while another study reported that the percentages of CXCR5^+^Foxp3^+^CD25^+^ cTfr cells and the ratio of Tfr/Tfh were decreased in SS patients (78).

The elevated enhancer of zeste homolog 2 (EZH2, an epigenetic regulator) in CD4^+^ T cells facilitated CXCR5^+^PD-1^+^ cTfh cell differentiation by enhancing STAT3 phosphorylation in SS patients (127). Elevated CCL25 expression in labial salivary glands could facilitate the attraction of circulating CCR9^+^ Th cells, which expressed high levels of PD-1 and ICOS in pSS patients. The CCR9^+^ Th cells promoted IgG production and displayed higher levels of IFN-γ, IL-17, IL-4, and IL-21 than CXCR5^+^ Th cells with antigen or IL-7 stimulation (74). Blocking ICOS reduced the levels of IL-21, IL-6, IL-8, and tumor necrosis factor-α (TNF-α) in SG–organ cultures, which indicated that T-cell costimulatory pathways were crucial for proinflammatory cytokine production of Tfh cells (76).

MSCs inhibited naive CD4^+^ T cells of SS patients to differentiate into cTfh cells via secreting indoleamine 2,3-dioxygenase (IDO) with high enzymic activity, which could be partly reversed by the IDO inhibitor 1-MT (128). Rituximab (RTX, B cell-depleting anti-CD20 monoclonal antibodies) therapy reduced cTfh cells in SS patients (129). Catalpol, sirolimus, and abatacept (CTLA-4-Ig fusion protein) therapy could reduce cTfh cells and upregulate cTfr cells and restore Tfh/Tfr ratio, which led to attenuate Tfh cell-dependent B-cell hyperactivity of SS (78, 130, 131).

## Granulomatosis with polyangiitis

GPA is a rare and severe systemic autoimmune disease with the classic hallmark of antineutrophilic cytoplasmic autoantibody (ANCA) specific for PR3 affecting systemic small vessels (132). Studies reported that TCR-activated naive CD4^+^ T cells from GPA patients expressed high levels of Bcl6, which was associated with decreased IL-2R/STAT5 signaling (133). CXCR5^+^PD-1^+^ cTfh cells (80) and CD4^+^IL-21^+^ IL-17A^+^ T cells were increased significantly in GPA patients, and ANCA-positive GPA patients had more CD4^+^IL-21^+^ and CD4^+^BCL6^+^ T cells in peripheral blood than HC and ANCA-negative GPA patients (81). RTX treatment decreased disease activity scores and cTfh cell percentages of active GPA patients (80).

## Multiple sclerosis

MS is a T cell-dominant chronic neuro-inflammatory disorder characterized by demyelination and axonal damage. Autoreactive CD4^+^ T cells from peripheral lymphoid organs or CD4^+^ T cells activated by central nervous system (CNS) local antigen play a crucial role in the pathogenesis of MS and its animal model, experimental autoimmune encephalomyelitis (EAE) (134). Genome-wide association studies (GWAS) showed that polymorphisms in the Tfh signature genes IL-21 (135), CXCR5 (136), and PD-1 (137) are either diagnostic or prognostic risk factors for MS.

Intrathecal inflammatory environment promoted the recruitment of cTfh cells (89), especially cTfh1 and CXCR3^+^CCR6^+^ cTfh17.1 cells into CNS (46), and CD4^+^IL-21^+^ T cells were found in the lesions of MS patients (82). The frequencies of CCR7^+^ICOS^+^ circulating memory Tfh cells, cTfh17 cells, and cTfh17.1 cells were increased and cTfh1 cells (83) and cTfh2 cells (84) were decreased significantly in MS patients (85–87). CXCR5^+^PD-1^+^ Tfh cells were also increased in the cerebrospinal fluid (CSF) of MS patients and EAE mice (88). Adoptive transfer of myelin antigen-activated splenic CXCR5^+^ Tfh cells exacerbated MS-like autoimmunity of EAE mice (138).

The frequencies of CXCR5^+^CD25^+^PD-1^+^Foxp3^+^/CD127^−^ or CXCR5^+^CD25^hi/+^ CD127^dim/−^ Tfr cells were decreased significantly and exhibited reduced suppressive capacity in blood and CSF of MS patients (85–87, 89). Blimp-1 deficiency impaired the suppressive activity and promoted the expression of pro-inflammatory cytokine IL17A in Tfr cells and their homing into the GC, which led to severe CNS autoimmunity in EAE mice (23, 139). The Tfr/Tfh ratio was decreased and negatively correlated with IgG production in serum and CSF of MS patients (87, 90).

Clinical studies showed that laquinimod treatment inhibited the expansion of PD-1^+^CXCR5^+^BCL6^+^ Tfh and IL-21-producing activated CD4^+^CD44^+^ T cells in the lymph nodes of EAE mice (140). Methylprednisolone pulse, abatacept, and RTX decreased cTfh cells and serum IL-21 in MS patients (141–143). Dimethyl fumarate treatment decreased the frequencies of cTfh1, cTfh17, and cTfh17.1 cells and increased cTfh2 cells in MS patients (84, 144). Fingolimod (sphingosine 1-phosphate receptor agonist) reduced frequencies of cTfh17, cTfh17.1, and CXCR5^+^CD25^hi^ cTfr cells, but increased cTfh1 cells in MS patients (145).

## Type 1 diabetes

T1D is a T cell-mediated organ-specific autoimmune disease. The pancreatic infiltrated islet-autoreactive T cells elicit hyperglycemia by destroying insulin-producing β cells (146). Unlike systemic autoimmune disease, Tfh cells are programmed differently in T1D. Although T–B cell interactions are essential to driving high-affinity islet autoantibody production predicting T1D development, the β-cell destruction can arise independently of autoantibody (91, 96, 147). Both Tfh and Tph cells were increased and associated with T1D progression in human and mouse models by producing IL-21 and recruiting and activating B cells in the pancreas (91–95). Furthermore, pathogenic Tfh1 cells were observed in the pancreas and promoted T1D development in nonobese diabetic (NOD) mice (96). Tfr cells were decreased and had attenuated suppressive ability in the peripheral blood, spleen, and pancreatic lymph nodes of T1D patients. The adoptive transfer of Tfr cells prevented T1D development in NOD mice (97, 98). RTX administration decreased the percentages of cTfh and CXCR5^+^PD-1^+^ cTfr cells but increased CXCR5^+^ICOS^+^ cTfr cells in T1D patients (148). Thus, Tfh cell analysis may be a biomarker and stratification tool to predict diabetes progression and clinical response for therapies in T1D patients (149, 150).

## Conclusions

Despite the fact that Tfh cells have been detected in many studies, their phenotypic surface markers vary in different studies. The expression of surface molecules and cytokines in Tfh cells changes over time to help B-cell responses more efficiently (10). In summary, Tfh cells are increased in multiple autoimmune diseases and promote the development of systemic autoimmune diseases by assisting B cell-mediated long-lasting humoral immunity (8). Islet autoantibodies are not thought to be the pathogenic effector molecules for T1D processes, but B cells, as antigen-presenting cells, can present islet autoantigen to active Tfh cells, which are necessary to cause β-cell destruction (96). Thus, the number of Tfh cells can be a novel predicting biomarker for clinical diagnosis and treatment of autoimmune diseases.

Tfr cells show different responses to distinct antigens or diverse disease contexts. The changes in Tfr cell percentages are not consistent in different studies on autoimmunity (10). Based on the discoveries to date, Tfr cells may also be induced and expanded by self-antigens, but their suppressive capacity is impaired in autoimmune diseases. The broken balance between Tfr and Tfh cells is responsible for the aggravated autoimmune responses. Targeting Tfr/Tfh balance may be a promising therapy for autoimmune diseases.

Theoretically, as a key transcription factor governing Tfh/Tfr differentiation, BCL6 degraders may be a potential therapeutic option by targeting Tfh cells in the treatment of autoimmune diseases. BCL6 is also an oncogenic driver for B-cell lymphoma and follicular lymphoma (151, 152). BCL6 targeting degraders have been well studied for lymphoma therapy (153, 154). Mechanistically, BCL6 contributes to lymphomagenesis by promoting the survival and proliferation of GC B cells and preventing premature terminal differentiation into memory or plasma cells, which can be beneficial for autoimmune diseases (151, 155). Hence, BCL6 degrader treatment may be a double-edged sword in autoimmune diseases and needs further basic and clinical research.

According to present clinical studies, methylprednisolone pulse, fingolimod, RTX, and other medication and biologics treatment decreased the expansion of pathogenic cTfh and/or increased cTfr cells in patients with autoimmune diseases (84, 117, 129, 141–145). However, some clinical studies showed unexpected results that the number of cTfh cells was not altered in RA patients treated with anti-TNFα agents (156) and the percentages of cTfh17 cells were increased in SLE patients treated with dexamethasone (111). Thus, further studies are required to better understand the delicate role of Tfh cell subsets in stratifying patients, which may help design personalized treatment schemes for individuals with autoimmune diseases.

## Author contributions

GY and JQ conceived and designed the project. JQ, CL, and ZB drafted and wrote the manuscript. JQ, GY, and XL supervised the project and revised the manuscript. All authors contributed to the article and approved the submitted version.
